# The probiotic *Lactobacillus kefiranofaciens* K6 alleviates exercise and sleep deprivation-induced physiological dysregulation and neuropsychiatric disorders via modulation of inflammation, circadian rhythm, and stress response

**DOI:** 10.7150/ijms.115387

**Published:** 2025-08-11

**Authors:** Chien-Wei Chen, Li-Ting Wu, Po-Hao Chiu, Yen-Po Chen, Wen-Ching Huang

**Affiliations:** 1 Department of Physical Education, Health, and Recreation, Teachers College, National Chiayi University, Chiayi 621302, Taiwan.; 2 Department of Exercise and Health Science, College of Human Development and Health, National Taipei University of Nursing and Health Sciences, Taipei 112303, Taiwan.; 3 Department of Educational Management, College of Education, National Taipei University of Education, Taipei 106320, Taiwan.; 4 Department of Animal Science, College of Agriculture and Natural Resources, National Chung Hsing University, Taichung 402202, Taiwan.

**Keywords:** probiotics, sleep deprivation, tight junction, sport nutrition, circadian rhythm

## Abstract

Individuals often suffer from insufficient or disrupted sleep due to night shifts, work pressure, and irregular lifestyles. Sleep deprivation (SD), defined as an intentional or unintentional reduction in sleep quality or quantity, has been associated with an increased risk of metabolic disorders, gut dysbiosis, emotional disturbances, and diminished performance in occupational and physical activities. Functional probiotics have been shown to regulate physiological homeostasis and ameliorate diseases through their impact on the microbiota and various physiological pathways. In this study, we employed the modified multiple platform method (MMPM) to induce SD in an animal model, simulating physiological dysregulation and psychological characteristics associated with SD. We further investigated whether exercise and the probiotic *Lactobacillus kefiranofaciens* K6 could mitigate the effects of SD on physiological homeostasis, neuropsychological function, inflammation, circadian rhythm, and exercise capacity. We found that the probiotic K6 significantly alleviated depression and anxiety while improving glucose intolerance and declining endurance capacity in the SD model. Elevated injury indexes (CK and LDH) induced by SD combined with exercise training were also significantly reduced under K6 supplementation. In the liver and muscle, SD alone or combined with exercise led to inflammation (*TNF-α*) and dysregulated circadian gene expression (*BMAL-1*,* CLOCK*), both of which were mitigated by K6 supplementation. In the intestine, hypothalamus, and hippocampus, SD or SD combined with exercise-induced inflammation (*TNF-α*, *IL-1β*) and tight junction hyperpermeability (*Claudin-1, ZO-1*) were alleviated with K6 supplementation, as were the circadian genes (*BMAL-1*, *CLOCK*) and corticotropin-releasing hormone receptor genes (*CRF1*, *CRF2*) in hypothalamus and hippocampus under SD alone or combined with exercise. The functional probiotic K6 improved physiological adaption, neuropsychological behaviors, and exercise performance with the implementation of SD and exercise training, potentially through regulation of inflammation, circadian rhythm, and stress response, contributing to overall health maintenance. The K6 probiotic strain may serve as a nutritional strategy to mitigate health risks and enhance performance affected by sleep deprivation.

## Introduction

Sleep is a highly complex physiological process essential in the management of physical and mental health. A population-based study revealed that over 40% of both men and women suffer from at least one sleep disorder 1. Further studies of adults across diverse nationalities found that 22%-53% of adults sleep less than 6 hours per night 2. The prevalence of poor sleep quality is becoming increasingly more common in modern society. From a physical and mental health perspective, insufficient sleep is positively associated with mood disturbances 3, cognitive impairment 4, cardiovascular disease mortality 5, and metabolic syndrome 6. The disruption of circadian rhythms and poor sleep quality associated with shift work may lead to impaired psychomotor performance, potentially due to hippocampal volume atrophy 7. In addition, the high prevalence of poor sleep among athletes, driven by time constraints, physical demands, and developmental requirements, may significantly impact performance, injury risk, cognitive function, and mental health 8. Given the numerous negative impacts of sleep restriction on health, addressing health risks from sleep loss could reduce healthcare demands and costs. Thus, researchers are continually investigating counteractive and preventive strategies for poor sleep quality or sleep deprivation (SD).

The meta-analysis investigating adults with sleep disorders or other sleep-related conditions found that probiotics may significantly improve sleep quality 9. Previous study has identified a correlation between gastrointestinal dysfunction and sleep disturbances, particularly in conditions such as irritable bowel syndrome (IBS) and functional dyspepsia 10. Probiotics may be regarded as psychobiotics, offering potential to alleviate gastrointestinal discomfort and stress-related symptoms while enhancing sleep quality 11. Animal studies have also demonstrated that the administration of probiotics may mitigate the adverse effects of SD-induced cognitive impairment and circadian rhythm disruption with modulation of gut microbiome and metabolite 12. The National Health and Nutrition Examination Survey also indicates that the consumption of yogurt and dietary supplements containing probiotics is associated with a reduced risk of sleep disturbances among U.S. adults, particularly males, individuals of white ethnicity, and those with a normal BMI 13. Despite these promising findings, the physiological mechanisms behind the effects of probiotics remain unclear. Understanding how probiotics influence physiological responses under SD conditions may help identify potential strategies for integrating them with other interventions.

Previous evidence-based studies demonstrate the physical activities and exercise intervention could not only provide benefits on health promotion, but also reduce the health risks on several chronic diseases and premature death 14-18. In modern society, lifestyles and work patterns are often dominated by sedentary habits, and work-related stress (long working hours) frequently leads to sleep deprivation, posing a significant hidden threat to health 19. Furthermore, extensive evidence suggests that exercise can alleviate the negative effects of SD and promote both physical and mental health. Scientific studies have highlighted the positive effects of exercise training on cognitive performance and its ability to attenuated blood pressure following partial or acute SD 20, 21. Preventive exercise may mitigate SD-induced cognitive impairment and anxiety-like behaviors through multiple pathways, including the regulation of oxidative stress, inflammation, neurotransmitters, and endocrine function 5. Sleep loss may also lead to a decline in sport-specific performance, immune system dysfunction, and autonomic nervous system imbalance, mimicking symptoms of overtraining syndrome 22. However, variations in exercise prescriptions, strategies, SD duration, and methods could influence the extent of physiological adaptation and disorders. To our knowledge, no studies have so far been conducted on the impact of exercise training and probiotic supplementation on physiological responses and physical activity following chronic SD (4 weeks). Therefore, the present study aims to investigate whether exercise and probiotic supplementation can alleviate the physiological dysregulation, exercise performance, and neuropsychiatric disorders associated with chronic SD.

The modified multiple platform method (MMPM) has been widely used to simulate sleep deprivation (SD) in rodents by disrupting both paradoxical (REM) and slow-wave sleep. This model reliably induces physiological changes such as elevated corticosterone levels, increased activity of hypocretin/orexin neurons, and fluctuations in melanin-concentrating hormone 23. Chronic SD is known to impair immune function, disrupt gut integrity, alter circadian rhythms, and negatively affect cognitive and physical performance—factors that mirror stress-related health challenges in humans. However, effective interventions to mitigate these multifaceted effects remain underexplored. Therefore, the present study aims to investigate whether the administration of *L. kefiranofaciens* K6 and regular exercise training can counteract SD-induced disruptions. Specifically, we examine their combined and individual effects on inflammation, intestinal permeability, neuropsychological behaviors, circadian rhythm, exercise performance, and physiological adaptations. We hypothesize that these interventions can attenuate the harmful consequences of chronic SD, providing insight into potential strategies for managing stress-related health risks in humans.

## Methods and Materials

### Experimental design

Six-week-old male C57BL/6 mice were obtained from the National Laboratory Animal Center (Taipei, Taiwan) and housed in the laboratory animal center at Taipei Medical University. The mice were maintained on a standard chow diet (No. 5001; PMI Nutrition International, Brentwood, MO, USA) and provided with sterile water *ad libitum* during the experiment. The environment was maintained under a 12-h light-dark cycle at 23°C ± 2°C and 60% ± 10% humidity. A veterinarian monitored the animals' behavior and disease status daily.

After a one-week period to acclimate to their diet and environment, the 7-week-old mice were allocated to five groups based on a normal distribution of body weight: Control, sleep deprivation (SD), sleep deprivation with exercise training (SD+Ex), sleep deprivation with *L. kefiranofaciens* K6 (SD+K6), and sleep deprivation with both exercise training and K6 (SD+Ex+K6). Before undergoing SD, the indicated groups received either probiotic supplementation and/or exercise training for 4 consecutive weeks. At 11 weeks of age, chronic SD was induced for 4 weeks (72 hours of sleep deprivation per week) in the SD, SD+Ex, SD+K6, and SD+Ex+K6 groups, with probiotic supplementation and/or exercise training continuing throughout this period. Behavioral analysis and oral glucose tolerance testing were conducted subsequently.

**Figure 1:**
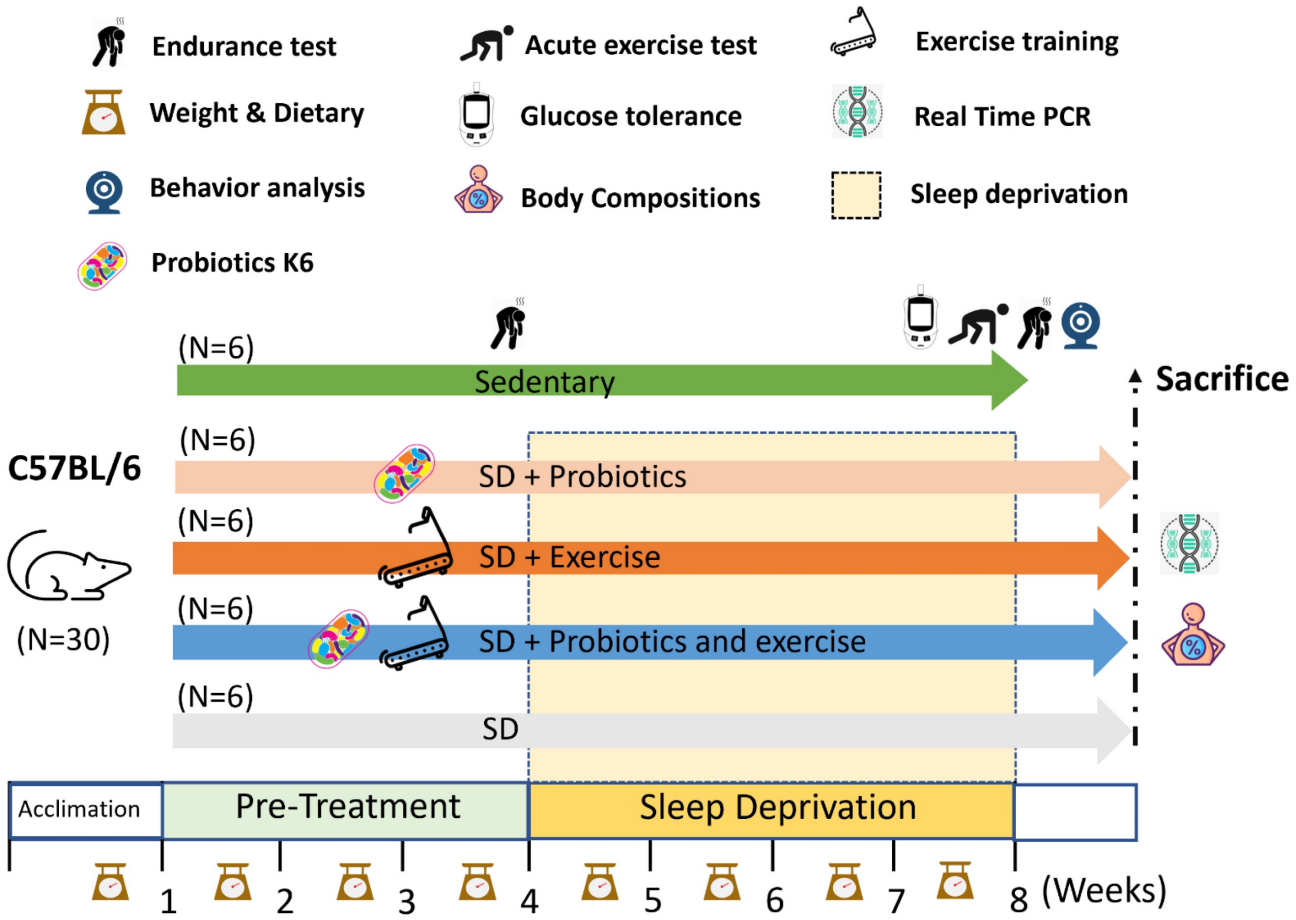
Experimental designs for the effects of exercise and probiotics supplementation in a sleep deprivation (SD) animal model. Animals were randomly assigned to one of five groups: Control, SD, SD+Ex, SD+K6, and SD+Ex+K6. Sleep deprivation was induced using the modified multiple platform method (MMPM) with 72 consecutive hours of deprivation per week for 4 weeks. Probiotics supplementation and exercise were administered to designated groups throughout the experimental period, including pre-treatment and sleep deprivation phases. Assessments took place within test duration and after sacrifice, and included inflammation, neuropsychological behaviors, glucose tolerance, exercise capacity, exercise injury, and the expression of genes related to circadian rhythm, tight junctions, and corticotropin-releasing factor receptors. K6:* Lactobacillus kefiranofaciens* K6; SD: sleep deprivation.

During the experiment, exercise performance was evaluated using the treadmill incremental load test (ILT) before and after SD. Peripheral fatigue-related biochemical variables were assessed through an acute exercise test. At the end of the study, mice were euthanized via asphyxiation with 95% CO_2_. Specifically, the optimal flow rate for CO₂ administration should be set to displace 10%-30% of the chamber volume per minute to ensure a humane and gradual induction. The organs of the mice, including the brain (hypothalamus and hippocampus), liver, muscle, kidney, spleen, heart, cecum, and epididymal fat, were collected, weighed, and subjected to further gene analysis (Figure 1). The experimental procedures in this study were approved by the Institutional Animal Care and Use Committee of Taipei Medical University (protocol number: LAC-2020-0055).

### Sleep deprivation induction

Chronic SD was induced in the mice by using a modified version of the MMPM, with a duration of 72 h per week, incorporating slight modification based on a previous study 24. Specifically, eight plastic platforms (diameter 3 cm × height 6 cm) were arranged at the bottom of a plastic tank (length 41 cm × width 21 cm × height 20 cm) and submerged in water to a depth of 1.0 cm below the platform's surface. This setup resulted in the mice falling into the water in order to disturb their sleep cycle, which took place after they achieved rapid eye movement (REM) sleep with muscle relaxation. The height of the platforms and the distance between them enabled the mice to move freely and access food. This murine model of SD effectively reduces slow-wave sleep and REM sleep 25. Body weight and dietary intake were monitored throughout the experiment.

### Probiotics supplementation

At 7 weeks of age, mice were administered the probiotic *L. kefiranofaciens* K6 via oral gavage for 8 consecutive weeks, including both pre-treatment and SD induction phases. The lyophilized probiotic powder was prepared at a concentration of 2.8 × 10^9^ CFU/g and stored at -20°C until use. For supplementation, the lyophilized powder was diluted in saline to achieve a dose of 8 × 10^7^ CFU/mouse/0.1 mL. Mice in the Control, SD, and SD+Ex groups received the equivalent volume of saline.

### Exercise training

The exercise training protocol was initially set at a speed of 10 m/min for 5 min, followed by 15 m/min for 30 min during the first week. In subsequent weeks, the training speed was adjusted and maintained at 12 m/min for 5 min and 18 m/min for 30 min.

### Exercise performance and associated biochemical variables in acute exercise test

Exercise performance was evaluated using the Incremental Load Test (ILT) before and after SD. The initial intensity of the ILT was set at 12 m/min with a 5% slope inclination, with the speed increased by 3 m/min every 3 min until exhaustion, as described in a previous study 22. For biochemical indexes associated with peripheral fatigue, the acute exercise protocol included 20 min of treadmill running at 18 m/min with no inclination. Blood was collected immediately after running for 20 min through submandibular venipuncture to measure the levels of lactate, ammonia (NH_3_), blood urea nitrogen (BUN), lactate dehydrogenase (LDH), and creatine kinase (CK).

### Oral glucose tolerance test

At the end of the SD intervention, the mice were fasted for 16 h before being orally administered glucose at a dose of 1.5 g/kg of body weight. Whole blood (0.6 μL) was sampled from the tail at the indicated time points: baseline, 15, 30, 60, and 120 min. This was performed using a glucometer (Accu-Chek^®^; Roche, Taipei, Taiwan) to measure plasma glucose fluctuations.

### Behavioral test

Following the indicated treatments, mouse behavior was evaluated using the elevated plus maze (EPM) test and tail suspension test (TST) to assess anxiety and depression levels. The detailed procedures for these behavioral tests are described in a previous study 26. Feces and urine were removed from the apparatus and the apparatus was cleaned using 75% ethanol before each subsequent test.

### Quantitative real-time polymerase chain reaction

Brain (hypothalamus and hippocampus), cecal, liver, and muscle tissues were extracted using the RNAzol RT kit (Molecular Research Center, OH, USA). Next, 1 μg of RNA was reverse transcribed into cDNA using the ToolsQuant II Fast RT Kit (BIOTOOLS, Taipei, Taiwan). Quantitative polymerase chain reaction (qPCR) was conducted using TOOLS 2× SYBR qPCR Mix (BIOTOOLS) on the Applied Biosystems QuantStudio 1 Real-Time PCR System (Thermo Fisher Scientific, Waltham, MA, USA). The cDNA templates were analyzed for the expression of inflammatory genes (*TNFA*, *IL1B*, and *IL6*), tight junction genes [occludin (*OCLN*), claudin-1 (*CLDN1*), and zonula occludens-1 (*TJP1*)], circadian genes (*BMAL1* and *CLOCK*), corticosteroid release factor receptors (*CRF1* and *CRF2*), and an internal control gene (*GAPDH*). The primers used to quantify gene expression are listed in Table 1. Gene expression was estimated using the threshold cycle values, and relative mRNA expression levels were calibrated using *GAPDH* expression.

### Statistical analysis

All data in this study are expressed as the mean ± standard deviation. Significant differences were analyzed using a one-way analysis of variance, and multiple between-group comparisons were performed using the *post hoc* Duncan test. The data were analyzed using SPSS Statistics (version 19.0; IBM, New York, NY, USA), and findings were considered statistically significant at *p* < 0.05.

## Results

### Growth curves, dietary intake

**Figure 2:**
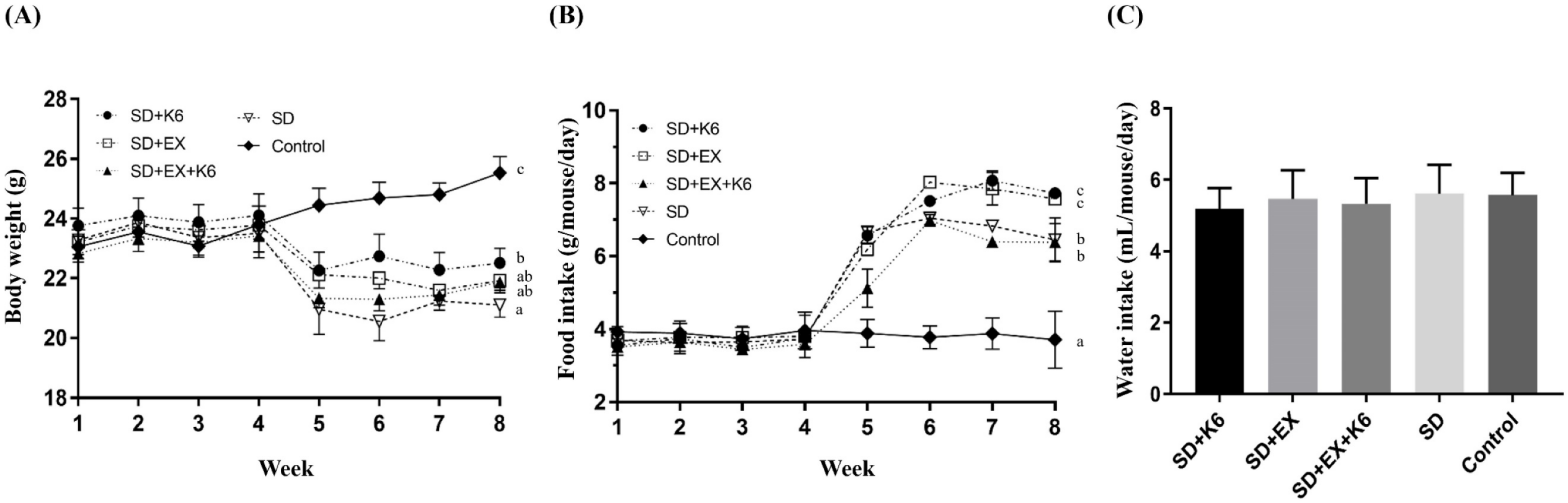
The effects of probiotic supplementation combined with exercise training on the growth curve (A) and dietary intake (food and water consumption) (B and C) under sleep deprivation conditions. Data are presented as the mean ± standard deviation. Columns labeled with different letters (a, b, and c) indicate significant differences between groups (*p* < 0.05) in a one-way analysis of variance.

During the pre-treatment phase with exercise and/or probiotics, there were no significant differences in body weight and food uptake across groups (F(4, 25) = 0.177-0.456,* p* > 0.05). After 4 weeks of SD induction in the SD+K6, SD+Ex, SD+Ex+K6, and SD groups, significant differences in body weight were observed (F(4, 25) = 15.78,* p* > 0.05) (Figure 2A). The SD groups had significantly lower body weights compared to the Control group. Notably, K6 supplementation in the SD+K6 group significantly ameliorated the SD-induced weight loss compared to the SD group. Regarding dietary intake, there was a significant increase in food intake in the SD groups compared to the Control group (F(4, 35) = 47.46,* p* < 0.001) (Figure 2B). The SD groups had higher food intake, with the SD+K6 group demonstrating greater dietary intake compared to the SD group. Water intake did not differ significantly among groups during the SD period (F(4, 35) = 0.485,* p* = 0.746) (Figure 2C).

### Exercise endurance performance

**Figure 3:**
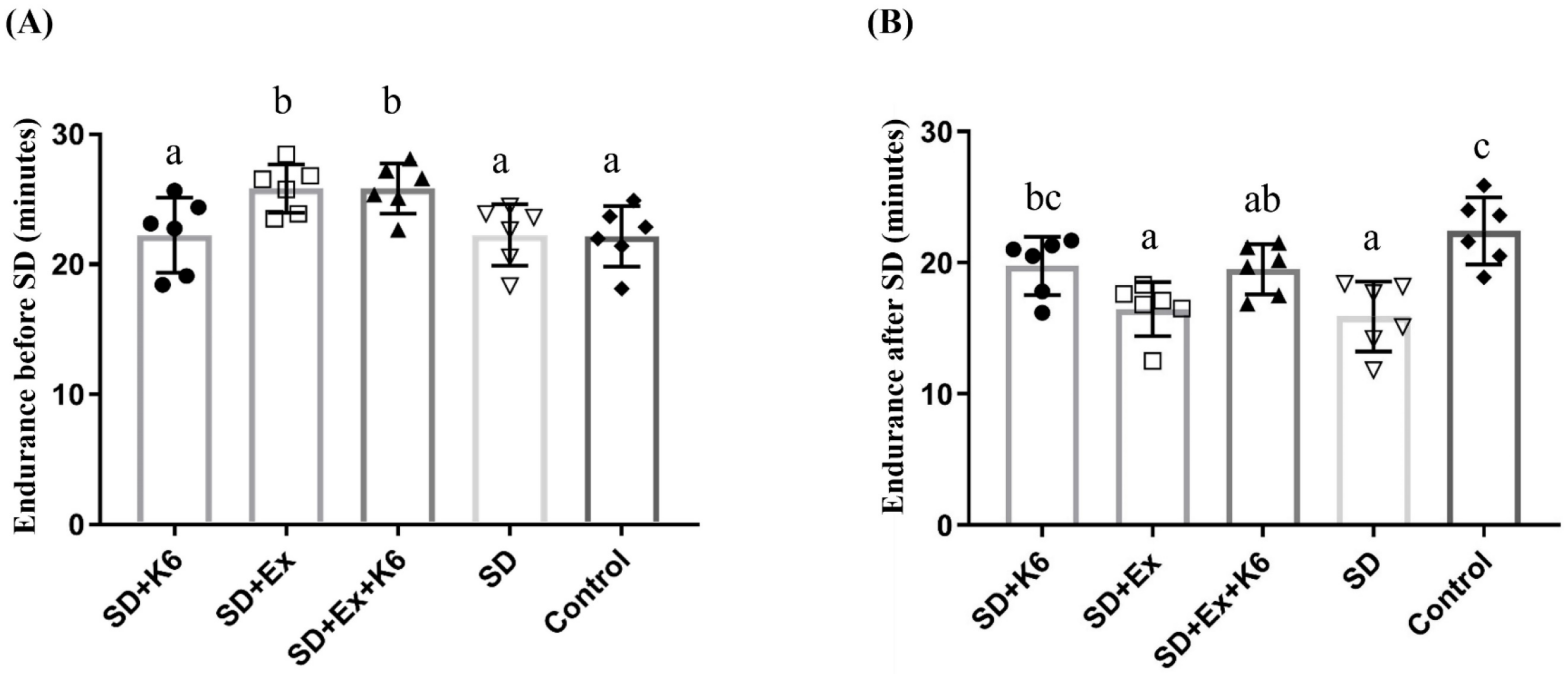
The effects of probiotic supplementation combined with exercise training on exercise performance under sleep deprivation conditions. (A) Endurance before SD, (B) Endurance after SD with the indicated treatments. Data are presented as the mean ± standard deviation. Columns labeled with different letters (a, b, and c) indicate significant differences between groups (*p* < 0.05) in a one-way analysis of variance.

Endurance was evaluated before and after SD using the ILT. Four weeks of pre-treatment with exercise and/or probiotics resulted in significant differences in endurance capacity among groups (F(4, 25) = 4.43,* p* = 0.008). Regular exercise training (SD+Ex and SD+Ex+K6) significantly improved endurance as compared to other groups (Figure 3A). After 4 weeks of SD, significant differences in endurance were observed among groups (F(4, 25) = 7.99,* p* = 0.0001) (Figure 3B). The SD and SD+Ex groups showed a significant decrease in endurance compared to the Control group. However, K6 supplementation (SD+K6) significantly alleviated the SD-induced endurance decrement compared to the SD group. Probiotic supplementation with K6 demonstrated more beneficial effects on mitigating endurance reduction than exercise alone in the SD model.

### Body compositions

Body composition was evaluated by measuring the weight of various tissues and organs at the end of the experiment (Table 2). No significant differences were observed in liver, spleen, kidney, muscle, and heart weights (F(4, 25) = 0.946-2.616,* p* > 0.05). However, the epididymal fat pad (EPF) and cecum displayed a significant difference among groups (F(4, 25) = 4.22-56.98,* p* < 0.05). Sleep deprivation (SD+K6, SD+Ex, SD+Ex+K6, and SD) led to a reduction in adipocyte tissue compared to the Control group, with K6 supplementation (SD+K6) significantly mitigating the SD-induced reduction. In addition, the weight of the cecum in the SD groups was significantly lower compared to the Control group, but there was no significant difference among the SD groups with K6 and/or exercise intervention.

### Glucose tolerance

**Figure 4:**
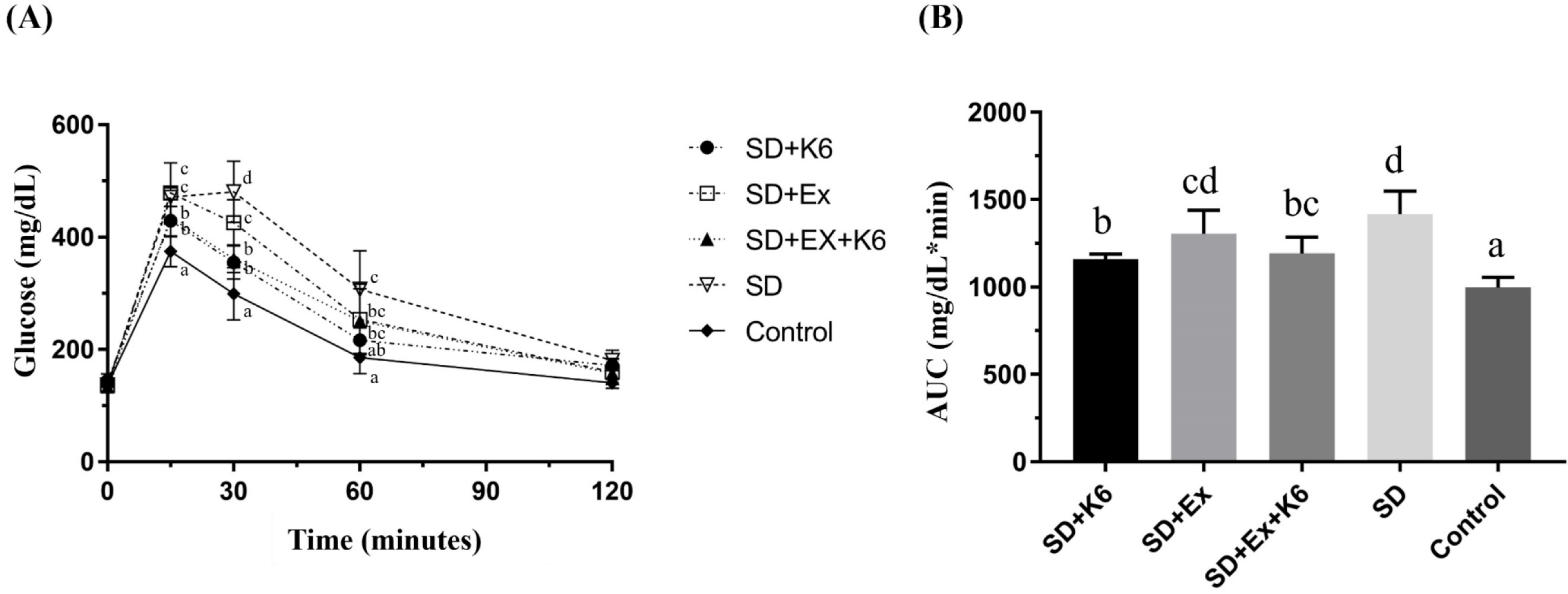
The effects of probiotic supplementation combined with exercise training on (A) the glucose tolerance test and (B) the area under the curve of oral glucose tolerance in chronic SD mice. Data are presented as the mean ± standard deviation. Columns labeled with different letters (a, b, c, and d) indicate significant differences between groups (*p* < 0.05) in a one-way analysis of variance.

Differences in blood glucose levels among groups were significant at the 15-, 30-, and 60-min time points (F(4, 25) = 4.62-17.51,* p* < 0.05) (Figure 4A). At the 15- and 30-min time points, blood glucose levels were significantly higher in the SD and SD+Ex groups compared to the K6 supplementation group (SD+K6 and SD+Ex+K6) and the Control group. At the 60-min time point, the SD group still showed significantly higher glucose levels compared to the SD+K6 and Control groups. Moreover, the area under the blood glucose change curve (Figure 4B) showed significant differences among groups (F(4, 25) = 15.64,* p* < 0.0001). The SD group exhibited greater glucose intolerance compared to the Control group in glucose area under curve. However, significant improvements in glucose tolerance were observed with K6 supplementation (SD+K6 and SD+Ex+K6) compared to the SD group. Overall, probiotics supplementation significantly improved glucose homeostasis under SD conditions.

### Behavior assessments

**Figure 5:**
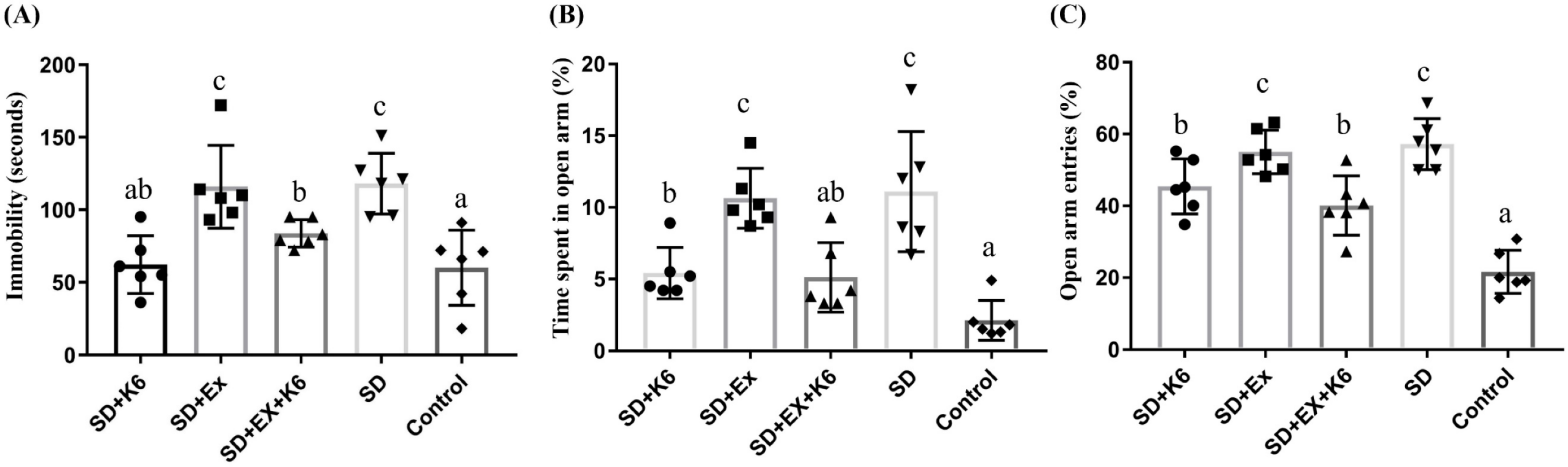
The effects of probiotic supplementation combined with exercise training on neuropsychological behaviors in chronic SD mice: (A) the TST and (B-C) the elevated plus maze test [(B) percentage of time spent exploring the open area and (C) percentage of entries into the open arm]. Data are presented as the mean ± standard deviation. Columns labeled with different letters (a, b, and c) indicate significant differences between groups (*p* < 0.05) in a one-way analysis of variance.

The TST is a mouse behavioral paradigm that measures depressive-like behaviors by assessing immobility time, with increased immobility indicating higher levels of depression 27. In the TST (Figure 5A), there were significant differences in immobility time among groups (F(4, 25) = 8.25,* p* < 0.0001), with immobility time found to be significantly higher in the SD groups (SD and SD+Ex) than in the Control group. However, K6 supplementation (SD+K6 and SD+Ex+K6) significantly reduced immobility time compared to the SD and SD+Ex groups, indicating a mitigation of depression with K6 supplementation.

The EPM test was used to assess anxiety levels post-SD 28. Significant differences were observed in both the percentage of time spent exploring the open area and the percentage of entries into the open arm (F(4, 25) = 13.63,* p* < 0.0001; F(4, 25) = 25.64,* p* < 0.0001, respectively) (Figure 5B and C). The SD groups (SD and SD+Ex) demonstrated a significantly higher amount of time spent and more entries into open arms compared to the Control group, indicating increased anxiety. In contrast, K6 supplementation (SD+K6 and SD+Ex+K6) significantly decreased both the time spent and number of entries into open arms compared to the SD and SD+Ex groups, reflecting significant reductions in anxiety-related behaviors due to probiotics supplementation.

### Biochemical variables after exercise intervention

**Figure 6:**
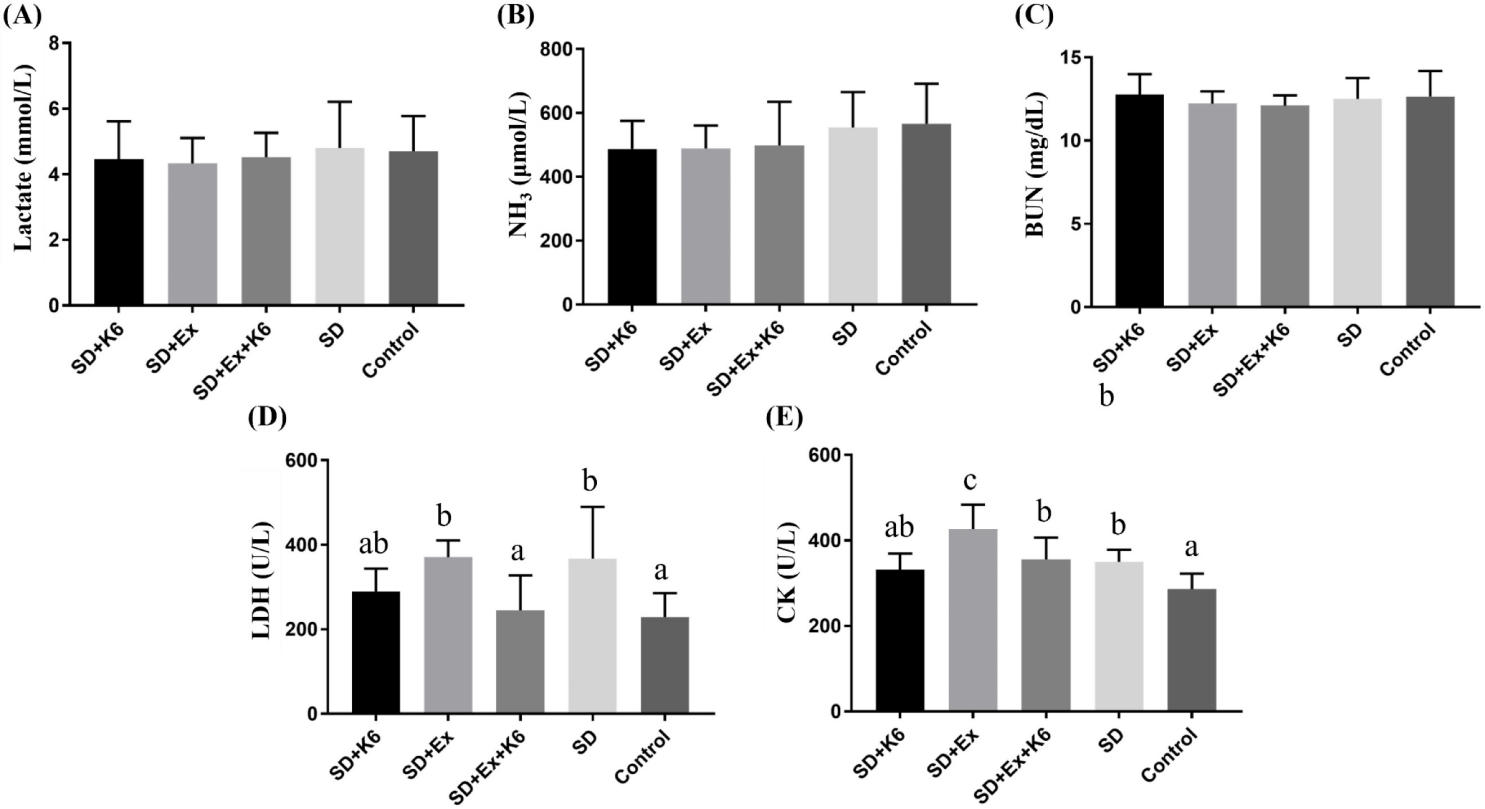
The effects of probiotic supplementation combined with exercise training on exercise biochemical variables in chronic SD mice: (A) Lactate, (B) NH3, (C) BUN, (D) LDH, and (E) CK after constant exercise intensity intervention. Data are presented as the mean ± standard deviation. Columns labeled with different letters (a, b, and c) indicate significant differences between groups (*p* < 0.05) in a one-way analysis of variance.

After acute exercise implementation, biochemical variables reflecting energy, metabolism, and injury related to exercise physiological adaptation were evaluated. No significant differences were observed among groups for lactate, ammonia, and BUN (F(4, 25) = 0.184-0.733,* p* > 0.05) (Figure 6). However, for injury-related indexes, significant difference were noted for LDH and CK among groups (F(4, 25) = 4.459,* p* = 0.007; F(4, 25) = 8.054,* p* < 0.0001, respectively). LDH levels were significantly higher in the SD and SD+Ex groups compared to the Control group, but K6 supplementation was found to mitigate the elevation, showing to significantly reduce LDH levels compared to the SD+Ex group. Similarly, CK levels were elevated in the SD+Ex group compared to the SD group, and K6 supplementation (SD+Ex+K6) significantly ameliorated the CK increase observed in the SD+Ex group.

### Inflammation and circadian gene expression in the liver and muscle

**Figure 7:**
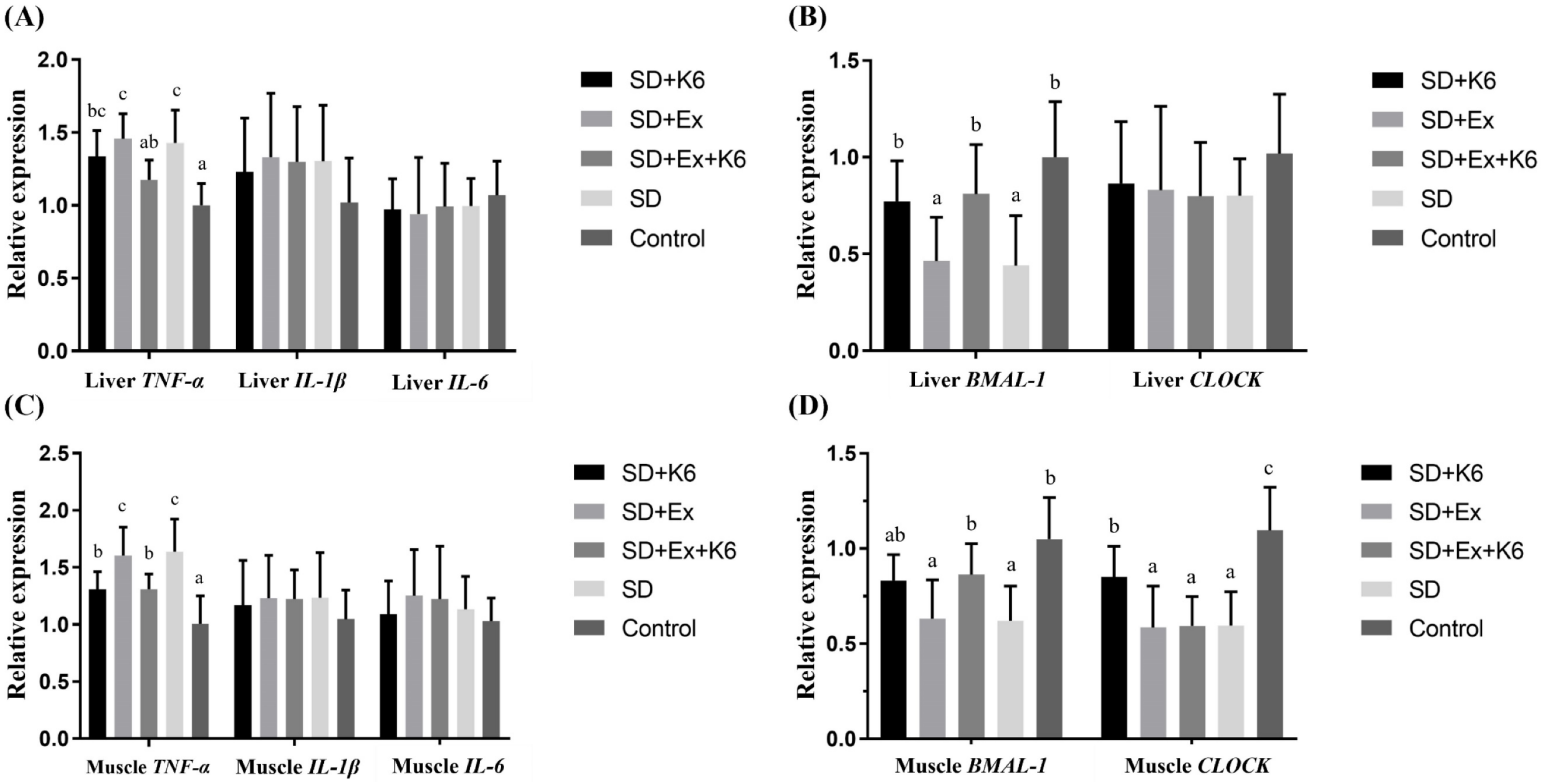
The effects of probiotic supplementation combined with exercise training on the expression of (A and C) inflammatory and (B and D) circadian genes in the liver (A and B) and the muscle (C and D) in chronic SD mice. Data are presented as the mean ± standard deviation. Columns labeled with different letters (a, b, and c) indicate significant differences between groups (*p* < 0.05) in a one-way analysis of variance.

Gene expression for inflammatory markers *TNFA*, *IL1B*, *IL6, and* circadian genes *BMAL1* and *CLOCK* was assessed in liver and muscle tissues. In the liver (Figure 7 A and B), no significant differences were found for *IL1B*, *IL6*, and *CLOCK* expression among groups (F(4, 25) = 0.179-0.687,* p* > 0.05). Significant differences were observed for *TNFA* and *BMAL1* expression (F(4, 25) = 5.579-7.164,* p* < 0.05). Both SD and SD+Ex groups showed significantly higher *TNFA* expression than the Control group. K6 supplementation (SD+Ex+K6) significantly reduced *TNFA* levels compared to the SD+Ex group. Additionally, SD and SD+Ex groups had significantly lower *BMAL1* expression than the Control group. The K6 supplementation (SD+K6 and SD+Ex+K6 significantly modulated the *BMAL1* expression as compared to SD and SD+Ex groups, respectively.

In muscle tissue (Figure 7 C and D), there was no significant differences in the expression of inflammatory genes (*IL1B*, and *IL6*) (F(4, 25) = 0.329-0.436,* p* > 0.05). However, *TNFA* and circadian genes (*BMAL1* and *CLOCK*) showed significant differences among the groups (F(4, 25) = 5.705-8.558,* p* < 0.05). *TNFA* expression was higher in the SD and SD+Ex groups compared to the Control group, but K6 supplementation (SD+K6 and SD+Ex+K6) significantly reduced *TNFA* levels compared to the SD and SD+Ex groups. *BMAL1* and *CLOCK* gene expressions were significantly lower in the SD and SD+Ex groups compared to the Control group. The expression of *BMAL1* was significantly higher in the SD+Ex+K6 group than the SD+Ex group, while *CLOCK* was more strongly expressed in the SD+K6 group as compared to the SD group, but still significantly lower than in the Control. These findings suggest that probiotic supplementation can modulate inflammation and circadian rhythm disruptions caused by sleep deprivation, contributing to physiological homeostasis.

### Inflammation, tight junction, and circadian gene expression in the intestine

**Figure 8:**
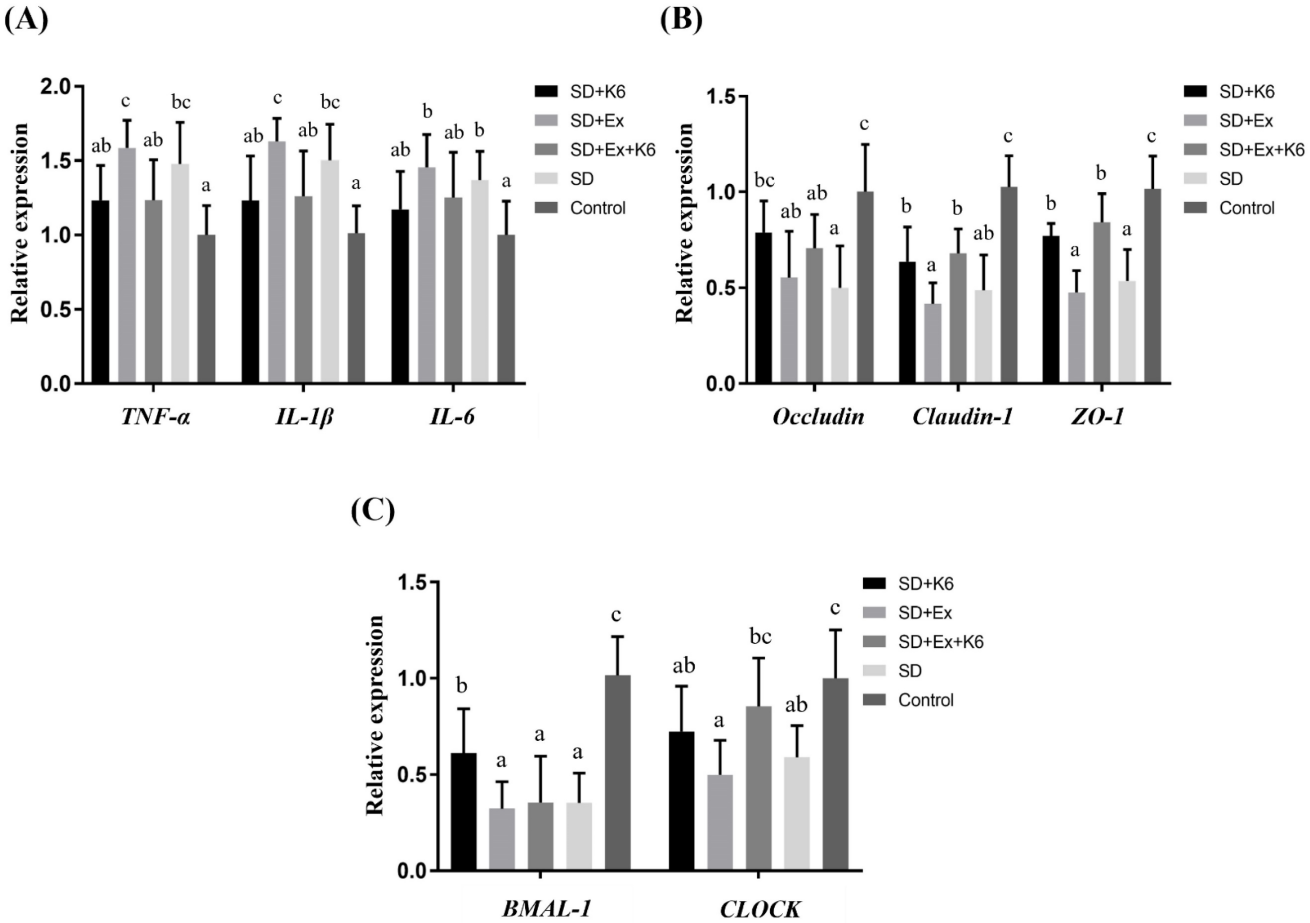
The effects of probiotic supplementation combined with exercise training on (A) intestinal inflammation, (B) tight junction, and (C) circadian rhythm in chronic SD mice. Data are presented as the mean ± standard deviation. Columns labeled with different letters (a, b, and c) indicate significant differences between groups (*p* < 0.05) in a one-way analysis of variance.

The expression of* TNFA*, *IL1B*, *IL6*, *OCLN*, *CLDN1*, *TJP1*, *BMAL1*, and *CLOCK* genes were analyzed in the proximal large intestine (cecal) tissue. A significant difference was observed in inflammation (*TNFA*, *IL1B*, *IL6*), tight junction (*OCLN*, *CLDN1*, *TJP1*), and circadian genes (*BMAL1*, and *CLOCK*) (F(4, 25) = 3.134-15.525,* p* < 0.05) (Figure 8). The SD and SD+Ex groups revealed significantly higher gene expressions for *TNFA*, *IL1B*, and *IL6* compared to the Control group. It was demonstrated that probiotics supplementation was able to alleviate inflammation, as gene expression of *TNFA* and *IL1B* were lower in the SD+Ex+K6 group as compared to the SD+Ex group. It was found that SD can induce intestinal hyperpermeability, as downregulation was observed in tight junction genes *OCLN*, *CLDN1*, and *TJP1* in the SD and SD+Ex groups as compared to the Control. The effects of probiotics supplementation on SD (SD+K6) showed a significant improvement on *ZO-1* and *OCLN* tight junctions, as compared to the SD group. The downregulation of *CLDN1* and *ZO-1*, induced from sleep deprivation and exercise training (SD+Ex), could be modulated by probiotic supplementation, as shown in the SD+Ex+K6 group. A significant decrease in the circadian genes *BMAL1* and *CLOCK* was observed in the SD and SD+Ex groups, as compared to the Control. These results show that probiotic supplementation could significantly modulate *BMAL1* and *CLOCK* expression, as demonstrated by the difference in their expression in the SD+K6 and SD+Ex+K6 groups compared to the SD and SD+Ex groups.

### Inflammation, tight junction, and circadian gene expression in the hypothalamus

**Figure 9:**
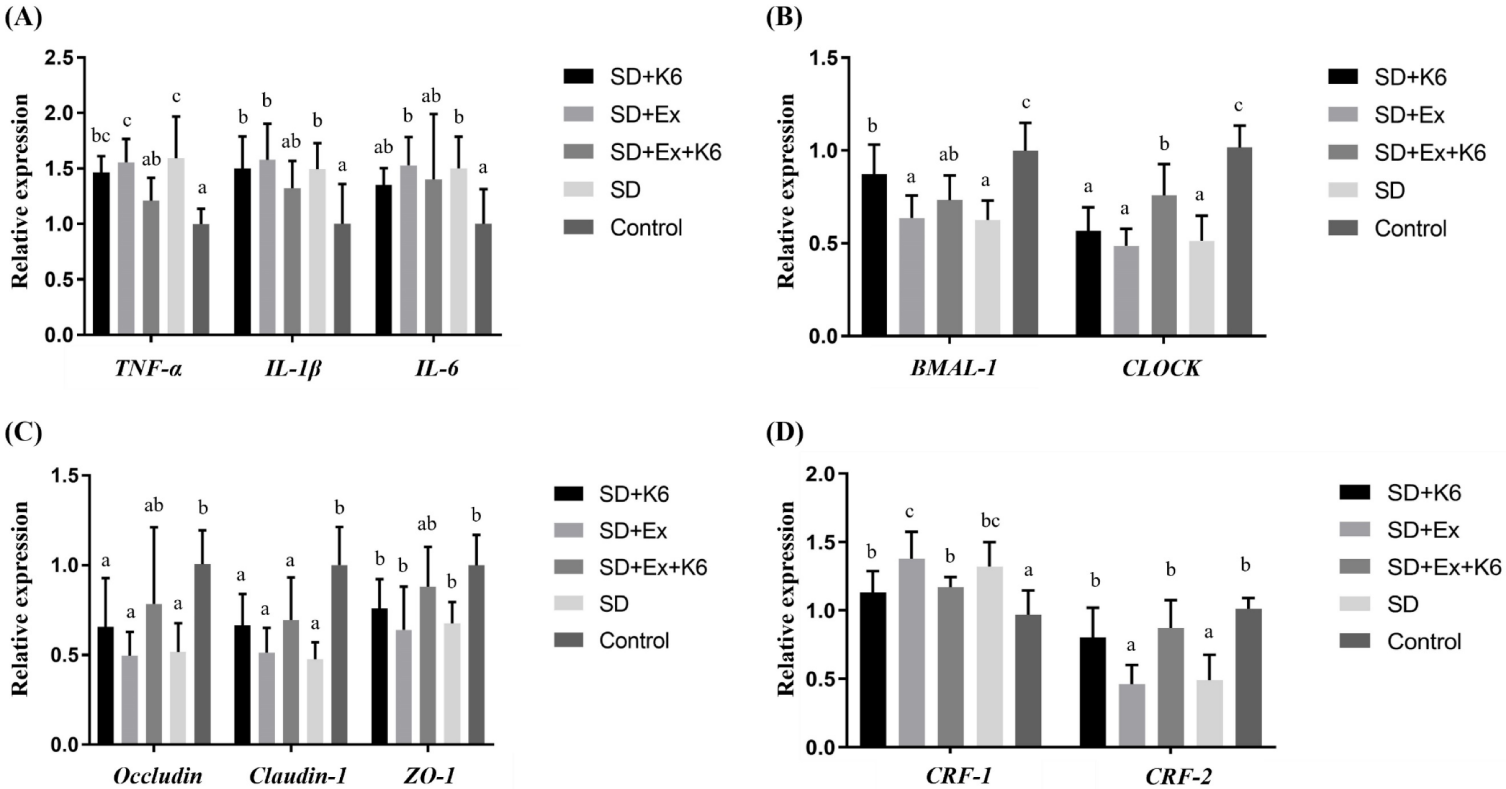
The effects of probiotic supplementation combined with exercise training on (A) inflammation, (B) circadian rhythm, (C) tight junction, and (D) corticotropin-releasing hormone receptors in the hypothalamus of chronic SD mice. Data are presented as the mean ± standard deviation. Columns labeled with different letters (a, b, and c) indicate significant differences between groups (*p* < 0.05) in a one-way analysis of variance.

In hypothalamus tissue, significant differences were observed in the expression of inflammation genes (*TNFA, IL1B,* and *IL6*) among groups (F(4, 25) = 3.681-7.106,* p* < 0.05) (Figure 9A). Sleep deprivation (SD and SD+Ex) significantly increased the levels of these inflammation genes compared to the Control group. K6 supplementation (SD+Ex+K6) significantly decrease the levels of the *TNFA* gene in the SD+Ex group. For circadian genes (*BMAL1* and *CLOCK*), significant differences were observed among groups (F(4, 25) = 8.565-17.147,* p* < 0.05) (Figure 9B). Both *BMAL1* and *CLOCK* levels were significantly reduced in the SD and SD+Ex groups as compared to the Control group. K6 supplementation significantly increased *BMAL1* and *CLOCK* expression in the SD+K6 and SD+Ex+K6 groups, respectively, compared to the SD and SD+Ex groups. Among tight junction genes (*OCLN*, *CLDN1*, *TJP1*) (Figure 9C), significant differences were observed among groups (F(4, 25) = 3.786-8.075,* p* < 0.05). SD led to a significant decrease in the expression of these genes in the SD, SD+Ex, and SD+K6 groups as compared to the Control. K6 supplementation did not show significant regulation of tight junction genes between groups. Regarding the expression of the corticotropin-releasing hormone receptor genes (*CRF1* and *CRF2*) in hypothalamus (Figure 9D), significant difference were observed (F(4, 25) = 6.16,* p* = 0.001; F(4, 25) = 11.857,* p* < 0.0001). SD and SD+Ex treatments significantly increased *CRF1* expression compared to the Control group. K6 supplementation (SD+Ex+K6) significantly reduced CRF1 levels compared to the SD+Ex group. Conversely, *CRF2* expression was significantly downregulated in the SD and SD+Ex groups compared to the Control. K6 supplementation notably improved *CRF2* expression in the SD+K6 and SD+Ex+K6 groups compared to the SD and SD+Ex groups, respectively.

### Inflammation, tight junction, and circadian gene expression in the hippocampus

**Figure 10:**
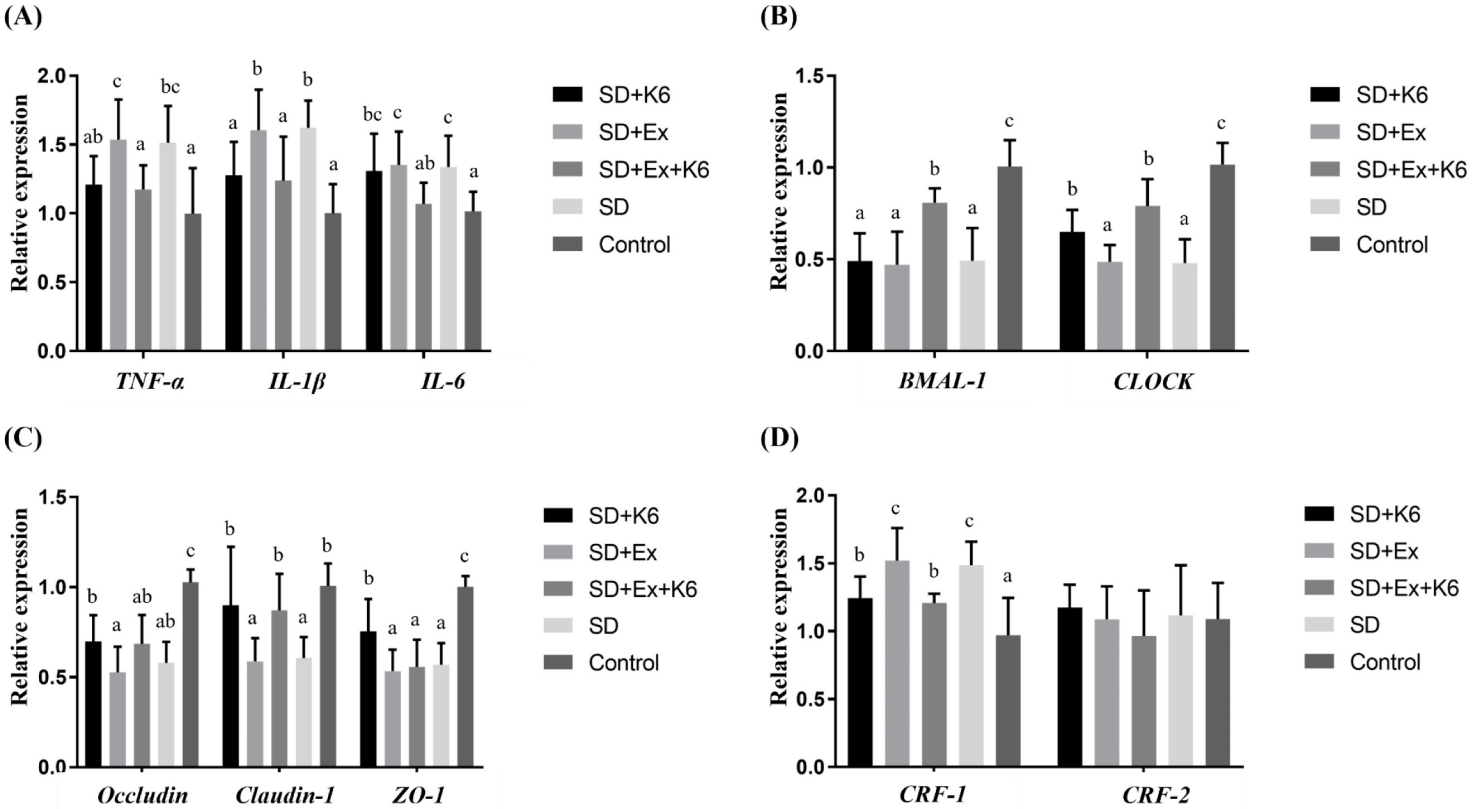
The effects of probiotic supplementation combined with exercise training on (A) inflammation, (B) circadian rhythm, (C) tight junction, and (D) corticotropin-releasing hormone receptors in the hippocampus of chronic SD mice. Data are presented as the mean ± standard deviation. Columns labeled with different letters (a, b, and c) indicate significant differences between groups (*p* < 0.05) in a one-way analysis of variance.

The inflammation gene expression (*TNFA*, *IL1B*, and* IL6*) in the hippocampus tissue demonstrated significant differences among groups (F(4, 25) = 3.402-6.287,* p* < 0.05) (Figure 10A). Both SD and SD+Ex groups exhibited significantly higher levels of these inflammation genes compared to the Control. K6 supplementation (SD+Ex+K6) significantly modulated the expression of *TNFA*, *IL1B*, and* IL6* compared to the SD+Ex group, with the SD+K6 group showing a significant decrease in *IL1B* expression compared to the SD group. Circadian genes (*BMAL1* and *CLOCK*) also showed significant differences among groups (F(4, 25) = 15.259-20.472,* p* < 0.05) (Figure 10B). SD led to significant downregulation of these genes in the SD and SD+Ex groups compared to the Control. K6 supplementation in the SD+Ex+K6 group significantly alleviated the decline of these circadian genes (*BMAL1* and *CLOCK*) compared to the SD+Ex group. Notably, the SD+K6 group also showed a significant increase in *CLOCK* gene expression compared to the SD group. As for tight junction gene expression (*OCLN*, *CLDN1*, *TJP1*), significant differences were observed among groups (F(4, 25) = 5.451-13.493,* p* < 0.05) (Figure 10C). The *CLDN1* gene*,* downregulated by sleep deprivation in the SD and SD+Ex groups, showed significant upregulation with K6 supplementation (SD+K6 and SD+Ex+K6). In addition, the *TJP1* gene was significantly regulated in the SD+K6 group compared to the SD group. Regarding corticotropin-releasing hormone receptor genes (Figure 10D), significant differences were observed in *CRF1* expression (F(4, 25) = 7.812,* p* < 0.001), but not in *CRF2* expression (F(4, 25) = 0.555,* p*= 0.679). Sleep deprivation was shown to activate *CRF1* gene expression in the SD and SD+Ex groups, while K6 supplementation significantly reduced CRF1 levels in the SD+K6 and SD+Ex+K6 groups.

## Discussion

We here used MMPM to induce chronic sleep deprivation (SD) in a murine model to investigate whether exercise training and probiotic K6 supplementation could alleviate the physiological maladaptation and neuropsychiatric disorders associated with chronic SD. Regular exercise training did not mitigate the adverse impacts of SD on inflammation, neuropsychological disorders, glucose intolerance, exercise performance decrement, or circadian rhythm dysregulation. In fact, it may have exacerbated exercise-associated injury indexes (CK) immediately after exercise. However, probiotic K6 supplementation improved exercise-induced injury indexes (LDH and CK) in the SD+Ex+K6 group compared to the SD group. In addition, K6 probiotics significantly mitigated SD-induced weight loss, exercise performance decrement, glucose intolerance, inflammation (*TNF-α*, *IL-1β*), tight junction hyperpermeability (*Claudin-1, ZO-1*), and circadian dysregulation (*BMAL-1*,* CLOCK*). Probiotic K6 also alleviated neuropsychiatric disorders induced by SD through modulation of the hypothalamic-pituitary-adrenal axis (*CRF1* and *CRF2*). Thus, probiotics supplementation may be a promising treatment for the prevention of health risks associated with sleep deprivation, especially when combined with exercise training.

Previous studies using the MMPM model demonstrated that both acute 72-hour sleep deprivation (SD) and chronic SD resulted in significantly higher food intake and inflammation, along with notable changes in body composition, including significantly lower body weight, in the SD group compared to the control group 26, 29. For the relation between inflammation and adipocyte tissue, the inflammation-induced lipolysis may be mediated by inositol-requiring protein 1 (IRE1) in adipocytes, independent of adipocyte insulin resistance 30. In addition, the study also revealed that SD could induce an increase in lipolytic cytokines, such as IL-6 in adipose tissue, and elevate serum corticosterone levels, contributing to a reduction in fat mass 31. The body weight reduction is likely attributable to SD-induced inflammation and lipolysis, as evidenced by elevated inflammatory cytokines in adipose tissue and increased corticosterone levels, consistent with previous studies 30, 31. Notably, food intake was significantly higher in SD groups (Figure 2B), and mice had *ad libitum* access to food and water throughout the MMPM protocol, ruling out prolonged fasting as a cause of weight loss. K6 supplementation's ability to attenuate SD-induced weight loss highlights its potential to modulate energy metabolism and inflammation, contributing to improved physiological homeostasis. On the other hand, the sleep-wake behavior and circadian rhythms are closely interconnected with energy metabolism and dietary intake. Previous study have demonstrated that acute 6-hour sleep deprivation (SD) can increase dietary intake during the recovery period while reducing body weight, accompanied by dysregulation of clock gene expression 32. The different stress-induced chronic sleep disorders (7 days) could also significantly increase the appetite but not on body weight change 33. Moreover, chronic SD in the present study generated a similar trend in associated diet and body weight changes as compared to previous study 26. The development of an increasing number of experimental models has demonstrated that sleep deprivation influences the central regulation of appetite by modulating the expression and function of appetite-related hormones. The mechanisms underlying the interactions between various types of sleep deprivation and hormonal regulation require further investigation.

We created a murine model of chronic SD using the MMPM to investigate neuropsychological behavior. This type of model has been shown to engender neurobehavioral changes such as depressive- and anxiety-like behaviors 34. Furthermore, variations in behavioral tests, interventions, and the physical features of the maze used in the experiments can influence EPM behavior. Under specific conditions, these factors might lead to an alternative interpretation of anxiety, where increased open-arm exploration is indicative of heightened anxiety and a panic-like reaction to novel situations, contrary to the conventional interpretation 35. The transparent material of the elevated plus maze (EPM) could lead to varying interpretations of behavior; however, the observed anxiety-related behavior was consistent with findings from previous study 26. The tail-suspension test is a behavioral assay commonly employed to assess depressive-like states and to investigate the effects of various interventions on depression-related behaviors 27. The immobility time in the tail-suspension test exhibited a significant increase, indicating depressive-like behavior induced by chronic unpredictable mild stress. However, probiotics supplementation demonstrated the potential to alleviate depression, likely through the modulation of gene expression in the prefrontal cortex and alterations in gut microbiota composition 36. In the present study, depression and anxiety were also observed after 4 weeks of chronic sleep deprivation. The K6 probiotics significantly ameliorated SD-induced depression and anxiety, while exercise implementation did not yield such improvements.

The gut-brain axis plays a crucial role in influencing emotions, with gut microbiota having a significant impact. Research on probiotics targeting this connection is referred to as psychobiotics. This class of probiotics can produce and transmit neuroactive substances, such as γ-aminobutyric acid (GABA) and serotonin, which interact with the gut-brain axis. Possible mechanisms include anti-inflammatory effects and the ability to reduce the hypothalamic-pituitary-adrenal (HPA) axis activation, thereby alleviating depression and anxiety 37. Psychobiotics are also believed to play a key role in the regulation of affective disorders and the immune system, possibly by regulating the neuroimmune and physiological mechanisms involved in control axes, such as the HPA axis, the sympathetic-adrenal-medullary axis, and the inflammatory reflex 38. In our previous research, we also found that *L. plantarum* PS128 could alleviate symptoms of depression by improving inflammatory responses and regulating cortisol, dopamine, and serotonin levels 39. Additionally, a combination of probiotic* Bifidobacterium longum* OLP-01 together with exercise training was shown to significantly improve weight management, glucose tolerance, fat composition, exercise-related oxidative stress, and injury indexes (LDH, CK, and ALT) in a high-fat diet-induced obese murine model 40. Therefore, *L. kefiranofaciens* K6 could also be considered as a psychobiotic for modulating anti-inflammatory responses and HPA hyperactivation, potentially mitigating neuropsychiatric disorders, and serving as an ergogenic aid for exercise recovery and physiological adaptation.

Circadian rhythm and inflammation are mutually influential. The macrophage-intrinsic circadian clock can be influenced by lipopolysaccharide-induced inflammatory responses through *BMAL1* suppression in macrophage immune cells, while inhibition of toll-like receptor 4 or nitric oxide show protection on circadian rhythms. Pro-inflammatory stimuli may lead to disrupted circadian rhythms, potentially contributing to cardiovascular diseases by altering macrophage behavior 36. Disruptions in the circadian clock due to sleep disturbance, light, or feeding times have been associated with inflammation and neurodegeneration, mediated by dysfunction in peripheral immune- and neuro-cells 41. A functioning circadian clock is also crucial for maintaining intestinal barrier integrity. A previous study reported that *BMAL1* deletion may dysregulate the circadian clock-related intestinal epithelium proliferation, inflammatory cytokines, and protein kinase activation in the gut. In addition, the pathogenesis of inflammatory bowel disease is influenced by the “biological clock” through its effects on intestinal barrier function 42. Rodent models have demonstrated the relationship between core clock genes and sleep/wake behavior, with *BMAL1* activity in the skeletal muscle being a pivotal regulator of *NREM* sleep duration 43. Thus, the present study suggests that sleep deprivation may induce intestinal inflammation and hyperpermeability, possibly through circadian rhythm disturbance, and that K6 probiotics can alleviate these impacts, either alone or when combined with exercise training.

With respect to the relationship between stress and corticotropin-releasing factor, systematic and emotional stimuli can induce a stress response in the hypothalamus as an initiation phase that involves activating the sympathetic-adrenal-medullary and HPA axes. This activation is typically regulated by a glucocorticoid negative feedback mechanism via the HPA axis. However, prolonged activation or dysregulation of this feedback mechanism may lead to health risks, including physiological diseases, psychological disorders, and pathogenesis 44. Corticotropin-releasing factor receptors (*CRFRs*) exhibit distinct expression patterns and functions in response to stress. The *CRF1* receptor is involved in the initial phases of stress response, characterized by quicker and more intense activation than *CRFR2*, and is associated with anxiety-related behaviors. Conversely, the *CRF2* receptor primarily participates in the later phase of the stress response, helping to counteract the activating effects of *CRFR1* and contributing to stress attenuation 45. Previous studies have shown that mice with a *CRF1* receptor gene defect exhibit reduced anxiety-like behaviors. Consequently, *CRF1* receptor antagonists have been explored as a treatment for anxiety-related symptoms through modulation of the HPA axis. Furthermore, research indicates that *CRF2*-null mice display increased anxiety-like behaviors 46, 47.

A review of sleep and performance in profession athletes has highlighted that athletes are particularly susceptible to sleep-related problems and disorders due to factors such as training, travel, competition factors, physical injury, illness, electronic device usage, and nutritional habits. Consequently, novel and accessible interventions should be developed for dynamic adaption within individualized training periodization 48. Insufficient sleep has been shown to be highly prevalent among athletes, and has negative impacts on injury risk, recovery, performance, and overall physical and mental health 8. Circadian rhythm dysregulation has also been association with mood disorders through HPA axis activation, as the circadian system influences the HPA axis, neurogenesis, melatonin, and monoaminergic neurotransmitters — factors that can contribute directly or indirectly to depression 49. Sleep deprivation has also been shown to exacerbate tissue injury and inflammation by reducing protein synthesis, thus negatively affecting physiological adaption 29. Insufficient sleep causes circadian misalignment, disrupting peripheral clocks and impairing skeletal muscle and liver metabolism, which ultimately disturbs overall energy homeostasis. Furthermore, fragmented or insufficient sleep disturbs the hormonal regulation, promoting a catabolic state that diminishes skeletal muscle protein synthesis rates 50. In the present study, K6 probiotics were found to mitigate exercise-related injuries and muscular inflammation, facilitating appropriate adaptation. Additionally, they were found to alleviate neuropsychiatric disorders, possibly by reducing HPA activation and modulating circadian genes.

## Conclusion

This study used the MMPM rodent model to simulate chronic sleep deprivation (SD) over the course of 4 weeks, which resulted in systemic inflammation, increased permeability, performance decrement, neuropsychiatric disorders, glucose intolerance, and HPA axis dysregulation. Furthermore, including exercise training in the SD model demonstrated physiological dysregulation similar to that of SD alone, with the addition of elevated tissue injury risk. In the case of sleep insufficiency, neither pretreatment nor combinatory exercise approaches seemed to improve physiological homeostasis, particularly under the conditions of SD. However, the probiotic K6 was effective in alleviating physiological dysregulation, neuropsychiatric disorders, and performance decline associated with SD, potentially through modulation of microbiota and associated physiological axes. When K6 supplementation was combined with exercise, it also showed promise in aiding in disease prevention and health promotion.

Individuals experiencing stress-associated sleep loss should carefully consider exercise intensity and exercise prescription to avoid potential physiological maladaptation and health risks. In addition, sleep quality and nutrition are crucial factors in athletes' health management, contributing to better adaptation in exercise training, psychological health, and physiological regulation. In high-pressure, fast-paced work environments and lifestyles, functional K6 probiotics could serve as a valuable nutritional strategy for alleviating stress-induced physiological dysregulation and neuropsychiatric disorders. Furthermore, a combination of probiotics supplementation, lifestyle management, and optimized exercise prescriptions may offer effective strategies for health promotion, functional maintenance, and disease prevention, particularly for shift workers, individuals in high-pressure working environment, those working in late-night establishments, and especially athletes.

## Figures and Tables

**Table 1:** The indicated genes primers applied to quantify genes expression in present study.

Genes	Primer sequences
*TNFA*	5′-CCACCACGCTCTTCTGTCTAC-3′ 5′-AGGGTCTGGGCCATAGAACT-3′
*IL1B*	5′-AGCTTCAGGCAGGCAGTATC-3′ 5′-TCATCTCGGAGCCTGTAGTG-3′
*IL6*	5′-CTTCCATCCAGTTGCCTTCTTG-3′ 5′-AATTAAGCCTCCGACTTGTGAAG-3′
*OCLN*	5′-TTGAAAGTCCACCTCCTTACAGA-3′ 5′-CCGGATAAAAAAGAGTACGCTGG-3′
*CLDN1*	5′-AGGTCTGGCGACATATTAGTGG-3′ 5′-CGTGGTGTTGGGTAAGAGGT-3′
*TJP1*	5′-GCACCATGCCTAAAGCTGTC-3′ 5′-ACTCAACACACCACCATTGC-3′
*BMAL1*	5′-ACATAGGACACCTCGCAGAA-3′ 5′-AACCATCGACTTCGTAGCGT-3′
*CLOCK*	5′-CCTATCCTACCTTCGCCACACA-3′ 5′-TCCCGTGGAGCAACCTAGAT-3′
*CRF1*	5′-CTACCAGGGCCCCATGATC-3′ 5′-CGGACAATGTTGAAGAGAAAGATAAA-3′
*CRF2*	5′-GGCAAGGAAGCTGGTGATTTG-3′ 5′-GGCGTGGTGGTCCTGCCAGCG-3′
*GAPDH*	5′-TGGTGAAGGTCGGTGTGAAC-3′ 5′-AATGAAGGGGTCGTTGATGG-3′

**Table 2:** Effects of probiotics combined with exercise training on body compositions in chronic SD mice.

Characteristic	SD+K6	SD+Ex	SD+Ex+K6	SD	Control
**Liver (g)**	1.03 ± 0.10	1.05 ± 0.12	0.96 ± 0.04	0.98 ± 0.05	1.01 ± 0.11
**Muscle (g)**	0.33 ± 0.03	0.30 ± 0.04	0.29 ± 0.03	0.28 ± 0.03	0.31 ± 0.04
**Kidney (g)**	0.33 ± 0.02	0.31 ± 0.01	0.29 ± 0.02	0.30 ± 0.04	0.33 ± 0.03
**Spleen (g)**	0.04 ± 0.02	0.04 ± 0.01	0.04 ± 0.01	0.05 ± 0.02	0.06 ± 0.03
**Heart (g)**	0.13 ± 0.01	0.13 ± 0.01	0.13 ± 0.01	0.13 ± 0.01	0.14 ± 0.02
**EPF (g)**	0.19 ± 0.03^b^	0.13 ± 0.01^a^	0.17 ± 0.01^ab^	0.15 ± 0.01^a^	0.34 ± 0.05^c^
**Cecum (g)**	0.35 ± 0.08^a^	0.34 ± 0.17^a^	0.37 ± 0.06^a^	0.38 ± 0.04^a^	0.55 ± 0.11^b^

Data are expressed as the mean ± SD in each group. Values in the same row with different superscript letters (a, b, c) differ significantly (p < 0.05) in a one-way analysis of variance (ANOVA). Muscle: gastrocnemius and soleus; EFP: epididymal fat pad. Control and HFD mean the normal chow diet and high fat diet administration, respectively.

## References

[1] McArdle N, Ward SV, Bucks RS, Maddison K, Smith A, Huang RC (2020). The prevalence of common sleep disorders in young adults: a descriptive population-based study. Sleep.

[2] Matsumoto T, Chin K (2019). Prevalence of sleep disturbances: Sleep disordered breathing, short sleep duration, and non-restorative sleep. Respir Investig.

[3] Short MA, Booth SA, Omar O, Ostlundh L, Arora T (2020). The relationship between sleep duration and mood in adolescents: A systematic review and meta-analysis. Sleep Med Rev.

[4] Fernandez-Mendoza J, He F, Puzino K, Amatrudo G, Calhoun S, Liao D (2021). Insomnia with objective short sleep duration is associated with cognitive impairment: a first look at cardiometabolic contributors to brain health. Sleep.

[5] Zhao B, Meng Y, Jin X, Xi W, Ma Q, Yang J (2023). Association of objective and self-reported sleep duration with all-cause and cardiovascular disease mortality: a community-based study. J Am Heart Assoc.

[6] Xi B, He D, Zhang M, Xue J, Zhou D (2014). Short sleep duration predicts risk of metabolic syndrome: a systematic review and meta-analysis. Sleep Med Rev.

[7] Song Y, Kim S, Joo Y, Ha E, Shim Y, Lee H (2024). Impact of sleep disturbance in shift workers on hippocampal volume and psychomotor speed. Sleep.

[8] Charest J, Grandner MA (2020). Sleep and athletic performance: impacts on physical performance, mental performance, injury risk and recovery, and mental health. Sleep Med Clin.

[9] Yu B, Wang KY, Wang NR, Zhang L, Zhang JP (2024). Effect of probiotics and paraprobiotics on patients with sleep disorders and sub-healthy sleep conditions: a meta-analysis of randomized controlled trials. Front Neurol.

[10] Kim SY, Choung RS, Lee SK, Choe JW, Jung SW, Hyun JJ (2018). Self-reported sleep impairment in functional dyspepsia and irritable bowel syndrome. J Neurogastroenterol Motil.

[11] Nishida K, Sawada D, Kawai T, Kuwano Y, Fujiwara S, Rokutan K (2017). Para-psychobiotic Lactobacillus gasseri CP2305 ameliorates stress-related symptoms and sleep quality. J Appl Microbiol.

[12] Tian P, Hou Y, Wang Z, Jiang J, Qian X, Qu Z (2024). Probiotics administration alleviates cognitive impairment and circadian rhythm disturbance induced by sleep deprivation. Food Sci Hum Wellness.

[13] Yang RZ, Lin SZ, Xie XY, Tang YJ, Zheng JX, Yuan CM (2024). Association between yogurt and dietary supplements containing probiotic consumption with sleep disturbance in US adults: Results from NHANES, 2009-2018. Heliyon.

[14] Sallis R, Young DR, Tartof SY, Sallis JF, Sall J, Li Q (2021). Physical inactivity is associated with a higher risk for severe COVID-19 outcomes: a study in 48440 adult patients. Br J Sports Med.

[15] Pedersen BK, Saltin B (2015). Exercise as medicine - evidence for prescribing exercise as therapy in 26 different chronic diseases. Scand J Med Sci Sports.

[16] Ekelund U, Tarp J, Steene-Johannessen J, Hansen BH, Jefferis B, Fagerland MW (2019). Dose-response associations between accelerometry measured physical activity and sedentary time and all cause mortality: systematic review and harmonised meta-analysis. BMJ.

[17] Dempsey PC, Friedenreich CM, Leitzmann MF, Buman MP, Lambert E, Willumsen J (2021). Global public health guidelines on physical activity and sedentary behavior for people living with chronic conditions: a call to Action. J Phys Act Health.

[18] Anderson E, Durstine JL (2019). Physical activity, exercise, and chronic diseases: A brief review. Sports Med Health Sci.

[19] Qiu D, Li Y, Li R, He J, Ouyang F, Luo D (2022). Long working hours, work-related stressors and sleep disturbances among Chinese government employees: A large population-based follow-up study. Sleep Med.

[20] Bommarito JC, Millar PJ (2024). Effects of aerobic exercise on ambulatory blood pressure responses to acute partial sleep deprivation: impact of chronotype and sleep quality. Am J Physiol Heart Circ Physiol.

[21] Taheri M, Irandoust K (2020). Morning exercise improves cognitive performance decrements induced by partial sleep deprivation in elite athletes. Biol Rhythm Res.

[22] Fullagar HH, Skorski S, Duffield R, Hammes D, Coutts AJ, Meyer T (2015). Sleep and athletic performance: the effects of sleep loss on exercise performance, and physiological and cognitive responses to exercise. Sports Med.

[23] Arthaud S, Varin C, Gay N, Libourel PA, Chauveau F, Fort P (2015). Paradoxical (REM) sleep deprivation in mice using the small-platforms-over-water method: polysomnographic analyses and melanin-concentrating hormone and hypocretin/orexin neuronal activation before, during and after deprivation. J Sleep Res.

[24] Patti CL, Zanin KA, Sanday L, Kameda SR, Fernandes-Santos L, Fernandes HA (2010). Effects of sleep deprivation on memory in mice: role of state-dependent learning. Sleep.

[25] Machado RB, Hipolide DC, Benedito-Silva AA, Tufik S (2004). Sleep deprivation induced by the modified multiple platform technique: quantification of sleep loss and recovery. Brain Res.

[26] Chung Y, Wu JL, Huang WC (2023). Effects of prebiotics on intestinal physiology, neuropsychological function, and exercise capacity of mice with sleep deprivation. Food Res Int.

[27] Can A, Dao DT, Terrillion CE, Piantadosi SC, Bhat S, Gould TD The tail suspension test. J Vis Exp. 2012: e3769.

[28] Walf AA, Frye CA (2007). The use of the elevated plus maze as an assay of anxiety-related behavior in rodents. Nat Protoc.

[29] Yang DF, Huang WC, Wu CW, Huang CY, Yang YSH, Tung YT (2023). Acute sleep deprivation exacerbates systemic inflammation and psychiatry disorders through gut microbiota dysbiosis and disruption of circadian rhythms. Microbiol Res.

[30] Foley KP, Chen Y, Barra NG, Heal M, Kwok K, Tamrakar AK (2021). Inflammation promotes adipocyte lipolysis via IRE1 kinase. J Biol Chem.

[31] Rosa Neto JC, Lira FS, Venancio DP, Cunha CA, Oyama LM, Pimentel GD (2010). Sleep deprivation affects inflammatory marker expression in adipose tissue. Lipids Health Dis.

[32] Dukanovic N, La Spada F, Emmenegger Y, Niederhauser G, Preitner F, Franken P (2022). Depriving mice of sleep also deprives of food. clocks sleep.

[33] Oishi K, Yajima Y, Yoshida Y, Hagihara H, Miyakawa T, Higo-Yamamoto S (2023). Metabolic profiles of saliva in male mouse models of chronic sleep disorders induced by psychophysiological stress. Sci Rep.

[34] Gonzalez-Castaneda RE, Galvez-Contreras AY, Martinez-Quezada CJ, Jauregui-Huerta F, Grcia-Estrada J, Ramos-Zuniga R (2016). Sex-related effects of sleep deprivation on depressive- and anxiety-like behaviors in mice. Exp Anim.

[35] Shoji H, Miyakawa T (2021). Effects of test experience, closed-arm wall color, and illumination level on behavior and plasma corticosterone response in an elevated plus maze in male C57BL/6J mice: a challenge against conventional interpretation of the test. Mol Brain.

[36] Xu J, Zhou L, Chen Z, Wang Y, Xu F, Kuang Q (2024). Bacillus coagulans and Clostridium butyricum synergistically alleviate depression in a chronic unpredictable mild stress mouse model through altering gut microbiota and prefrontal cortex gene expression. Front Pharmacol.

[37] Dinan TG, Stanton C, Cryan JF (2013). Psychobiotics: a novel class of psychotropic. Biol Psychiatry.

[38] Bermudez-Humaran LG, Salinas E, Ortiz GG, Ramirez-Jirano LJ, Morales JA, Bitzer-Quintero OK (2019). From probiotics to psychobiotics: live beneficial bacteria which act on the brain-gut axis. Nutrients.

[39] Liu YW, Liu WH, Wu CC, Juan YC, Wu YC, Tsai HP (2016). Psychotropic effects of Lactobacillus plantarum PS128 in early life-stressed and naive adult mice. Brain Res.

[40] Hsu YJ, Chiu CC, Lee MC, Huang WC (2021). Combination of treadmill aerobic exercise with Bifidobacterium longum OLP-01 supplementation for treatment of high-fat diet-induced obese murine model. Obes Facts.

[41] Wang XL, Li L (2021). Circadian clock regulates inflammation and the development of neurodegeneration. Front Cell Infect Microbiol.

[42] Stokes K, Cooke A, Chang H, Weaver DR, Breault DT, Karpowicz P (2017). The circadian clock gene BMAL1 coordinates intestinal regeneration. Cell Mol Gastroenterol Hepatol.

[43] Ehlen JC, Brager AJ, Baggs J, Pinckney L, Gray CL, DeBruyne JP (2017). Bmal1 function in skeletal muscle regulates sleep. Elife.

[44] de Kloet ER, Joels M, Holsboer F (2005). Stress and the brain: from adaptation to disease. Nat Rev Neurosci.

[45] Vasconcelos M, Stein DJ, Gallas-Lopes M, Landau L, de Almeida RMM (2020). Corticotropin-releasing factor receptor signaling and modulation: implications for stress response and resilience. Trends Psychiatry Psychother.

[46] Issler O, Carter RN, Paul ED, Kelly PA, Olverman HJ, Neufeld-Cohen A (2014). Increased anxiety in corticotropin-releasing factor type 2 receptor-null mice requires recent acute stress exposure and is associated with dysregulated serotonergic activity in limbic brain areas. Biol Mood Anxiety Disord.

[47] Kosari-Nasab M, Sadeghi T, Bashiri H, Shokouhi G, Salari AA (2019). The blockade of corticotropin-releasing factor 1 receptor attenuates anxiety-related symptoms and hypothalamus-pituitary-adrenal axis reactivity in mice with mild traumatic brain injury. Behav Pharmacol.

[48] Cook JD, Charest J (2023). Sleep and performance in professional athletes. Curr Sleep Med Rep.

[49] de Leeuw M, Verhoeve SI, van der Wee NJA, van Hemert AM, Vreugdenhil E, Coomans CP (2023). The role of the circadian system in the etiology of depression. Neurosci Biobehav Rev.

[50] Morrison M, Halson SL, Weakley J, Hawley JA (2022). Sleep, circadian biology and skeletal muscle interactions: Implications for metabolic health. Sleep Med Rev.

